# The role of inflammation in cerebral small vessel disease and vascular cognitive impairment, and therapeutic implications

**DOI:** 10.1177/17474930261461366

**Published:** 2026-06-08

**Authors:** Robin B Brown, Stuart M Allan, Hugh S Markus

**Affiliations:** 1 Department of Clinical Neurosciences, University of Cambridge, Cambridge, UK; 2 Division of Neuroscience, School of Biological Sciences, Faculty of Biology, Medicine and Health, The University of Manchester, Manchester, UK; 3 Geoffrey Jefferson Brain Research Centre, Manchester Academic Health Science Centre, Northern Care Alliance NHS Foundation Trust, The University of Manchester, Manchester, UK

**Keywords:** Leukoaraiosis, ischemic stroke, stroke, vascular cognitive impairment, vascular dementia, CADASIL, brain

## Abstract

**Background:**

Increasing evidence suggests that inflammation occurs in cerebral small vessel disease (SVD). Inflammation has been hypothesized to play a role in disease pathogenesis and also in progression to vascular cognitive impairment and dementia (VCID), suggesting that targeting inflammation may offer a novel treatment option.

**Aims:**

In this review, we aimed to summarize the evidence that both systemic and central nervous system (CNS) inflammation occur in SVD and VCID, critically examine whether associations are causal, and review the evidence that anti-inflammatory interventions might represent possible treatments. We include coverage of sporadic SVD, cerebral amyloid angiopathy, and genetic small vessel diseases.

**Summary of review:**

CNS inflammation has been demonstrated in SVD both in post-mortem brains and *in vivo* using positron emission tomography with radioligands targeted against the translocator surface proteins in microglia, but robust evidence showing such changes are causally related to disease progression is lacking. Peripheral inflammation can be measured in the blood using targeted assays and, more recently, large-scale proteomic panels. Proteins involved in coagulation, endothelial cell activation, and immune cell adhesion have been associated with SVD, as well as specific cytokines, although not all findings have been replicated, and there are limited data examining whether individual proteins predict future disease progression. Genetic data can be used to inform whether such associations are likely to be causal and prioritize treatment targets. However, to date, few treatment trials have investigated whether drugs that target specific inflammatory pathways can reduce SVD progression and onset of VCID, and those that have been performed have used non-specific inhibitors such as minocycline and colchicine.

**Conclusions:**

Considerable data support the presence of both systemic and CNS inflammation in SVD and VCID. However, whether these associations are causal remains unclear, and more longitudinal and interventional studies are required. A better understanding of the molecular basis of inflammation and immune dysfunction in SVD also allows more precise therapeutic targeting.

## Graphical abstract

**Graphical abstract:**
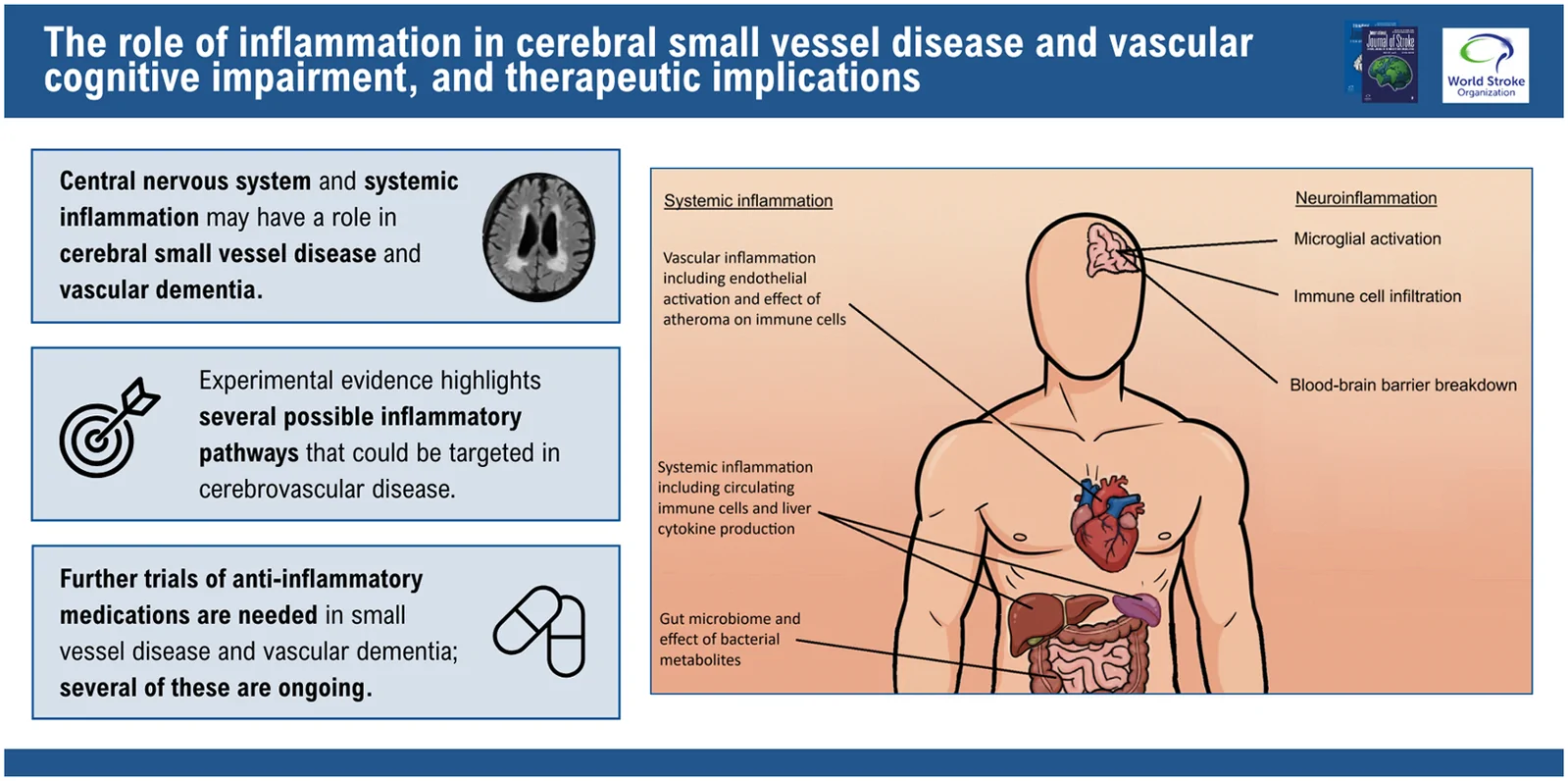

## Introduction

It is estimated, as of 2021, that around 56.9 million people worldwide are living with dementia. Approximately 15% of the dementia in individuals is caused by pure vascular dementia and 16% by mixed vascular and degenerative dementia.^1^ Various classifications have been proposed to describe cognitive impairment and dementia caused by cerebrovascular disease;^2,3^ for the purposes of this review, we refer to these as vascular cognitive impairment and dementia (VCID) as suggested in the recent International Society for Vascular Behavioral and Cognitive Disorders (VASCOG)—World Stroke Organization guidelines.^4^

Several cerebrovascular disease processes cause VCID, including single or multiple ischemic strokes and brain hemorrhage.^5^ The most common of these is cerebral small vessel disease (SVD),^6^ a pathological dysfunction in the penetrating arteries, arterioles, and venules within the brain that is often related to cardiovascular risk factors such as hypertension.^7^ SVD can affect predominantly white and deep gray matter (often assumed to be mediated by cardiovascular risk factors and termed sporadic SVD, or arteriolosclerosis), or through β-amyloid deposition in leptomeningeal vessels supplying cortical areas in cerebral amyloid angiopathy (CAA).^7^

Key radiological features include small subcortical (lacunar) infarcts, white matter hyperintensity lesions (WMHs), cerebral microbleeds (CMBs), and enlarged perivascular spaces (PVSs).^8^ These radiological features are often associated with VCID as SVD is responsible for around 45% of cases of VCID,^6^ although SVD pathology is also associated with increased prevalence and severity of other dementias, particularly Alzheimer’s disease (AD).^9^

There are no proven treatments for VCID,^10^ and therapeutic options in SVD are limited,^11^ although blood pressure reduction has been associated with a reduction in both WMH^12^ and dementia.^13^ This paucity of treatments has led to recent interest in targeting inflammation as a novel therapeutic approach. The immune system drives disease progression in other conditions, for example, atherosclerotic heart disease,^14^ leading to trials of immunomodulatory medication. For example, colchicine reduces recurrent cardiovascular events after cardiac ischemia^15,16^ and is now included in international guidelines for treatment after myocardial infarction.^17^

Both inflammation within the brain itself and systemic inflammation have been implicated in SVD and VCID,^18,19^ and Figure 1 shows the possible mechanisms that may contribute to this association. A key question is whether inflammatory processes are causal or merely secondary to tissue damage. In this review, we summarize the evidence that both systemic and central nervous system (CNS) inflammation occur in SVD, critically examine whether any associations are causal, and review the evidence that anti-inflammatory interventions might represent possible treatments.

**Figure 1.**
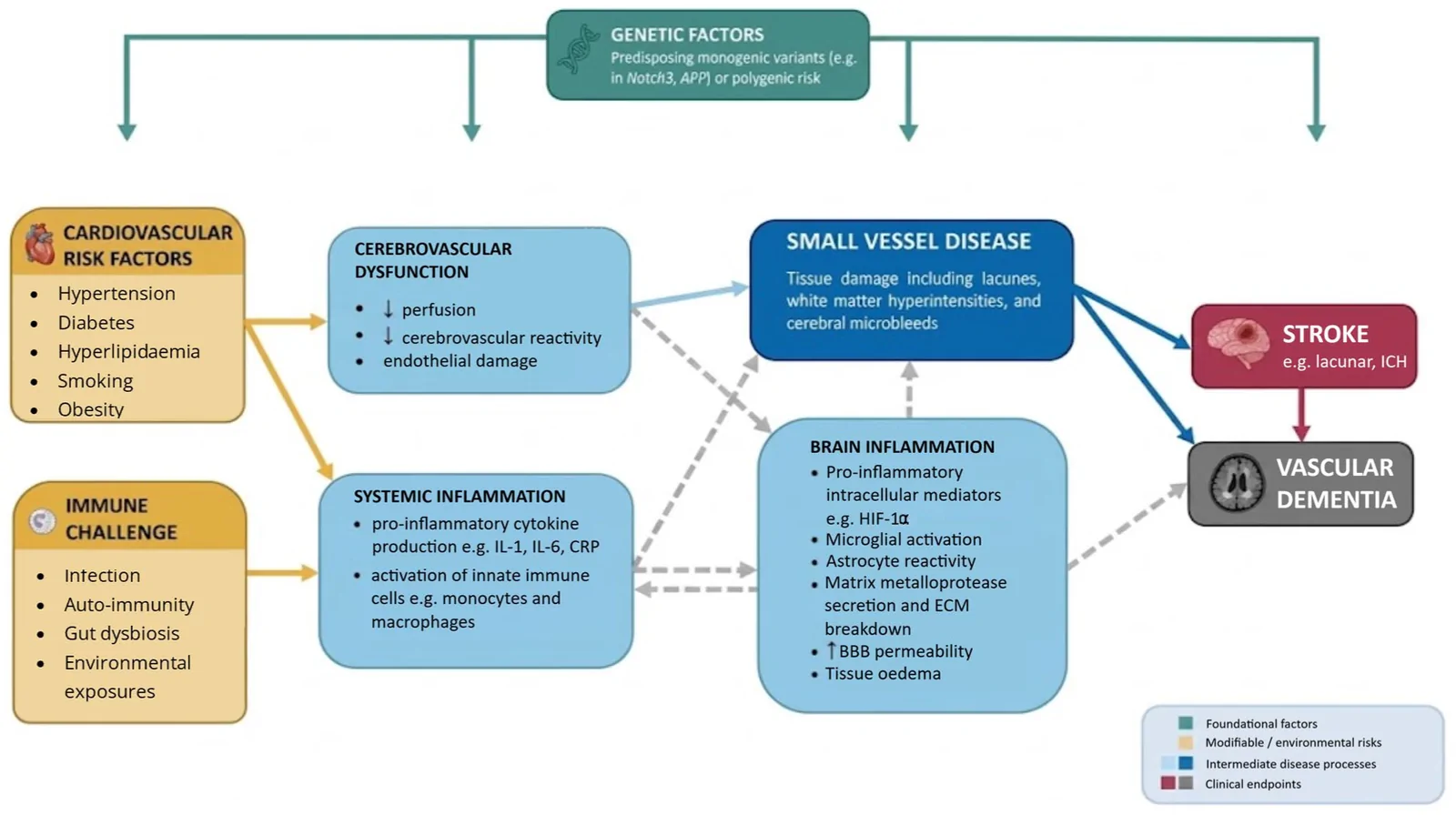
Schematic showing potential exposures and pathophysiological processes linked to the development of SVD and VCID. Consistent associations are shown as solid arrows; hypothesized associations or correlations in which the direction of causation is uncertain are shown by a dashed gray arrow. Genetic factors may be relevant at multiple stages, as there is potential for genetic variants to affect proteins involved in any of these processes. APP, amyloid precursor protein; BBB: blood–brain barrier; CRP: C-reactive protein; ECM: extracellular matrix; HIF-1α: hypoxia-inducible factor-1α; ICH: intracerebral hemorrhage; IL: interleukin.

## Methods

We performed a literature search using Ovid MEDLINE and Google Scholar to identify studies investigating the relationship between inflammation and SVD or VCID. The study was registered in the International Register of Prospective Systematic Reviews (PROSPERO; ID 1279164). Searches aimed to capture articles investigating SVD or VCID that assessed the following:

1. Evidence of CNS inflammation, including
  a. Neuropathological assessment of inflammatory markers or immune cell infiltration.
  b. Inflammation assessed in vivo using neuroimaging.
  c. Immune cell activity or inflammatory markers in cerebrospinal fluid (CSF).
2. Evidence of systemic inflammation, including
  d. Inflammatory blood biomarkers.
  e. Assessment of immune cell activity.

Search terms were as follows:

Ovid MEDLINE: “((vascular dementia or vascular cognitive impairment or cerebral small vessel disease or SVD or arteriolosclerosis or cerebral amyloid angiopathy or CAA or CADASIL) and (inflammation or immune)).”
Google Scholar: “vascular dementia or vascular cognitive impairment or cerebral small vessel disease or SVD or arteriolosclerosis or cerebral amyloid angiopathy or CAA or CADASIL and inflammation or immune.”

We included primary research articles published in English up to 30 November 2025, investigating inflammation in patients or control participants free from stroke or cognitive impairment. Articles from the reference lists of returned publications were also included. Studies investigating specific alternative causes of dementia (e.g. AD) or patients with unselected stroke were excluded as these may have different mechanisms. Abstracts were screened by one author (RBB) before full-text review, and included articles were reviewed for relevance by the second author (HSM). Any uncertainties were resolved by discussion.

## Results

A total of 2007 abstracts were screened, and 327 were selected for full-text review (Figure 2). These studies included 34 neuropathological studies, 182 articles investigating blood markers of inflammation, 32 genetic studies, 11 imaging experiments, and 14 studies investigating CSF. Forty-three studies investigated the association of SVD/VCID with inflammatory comorbid conditions, including infections and autoimmune disease.

**Figure 2.**
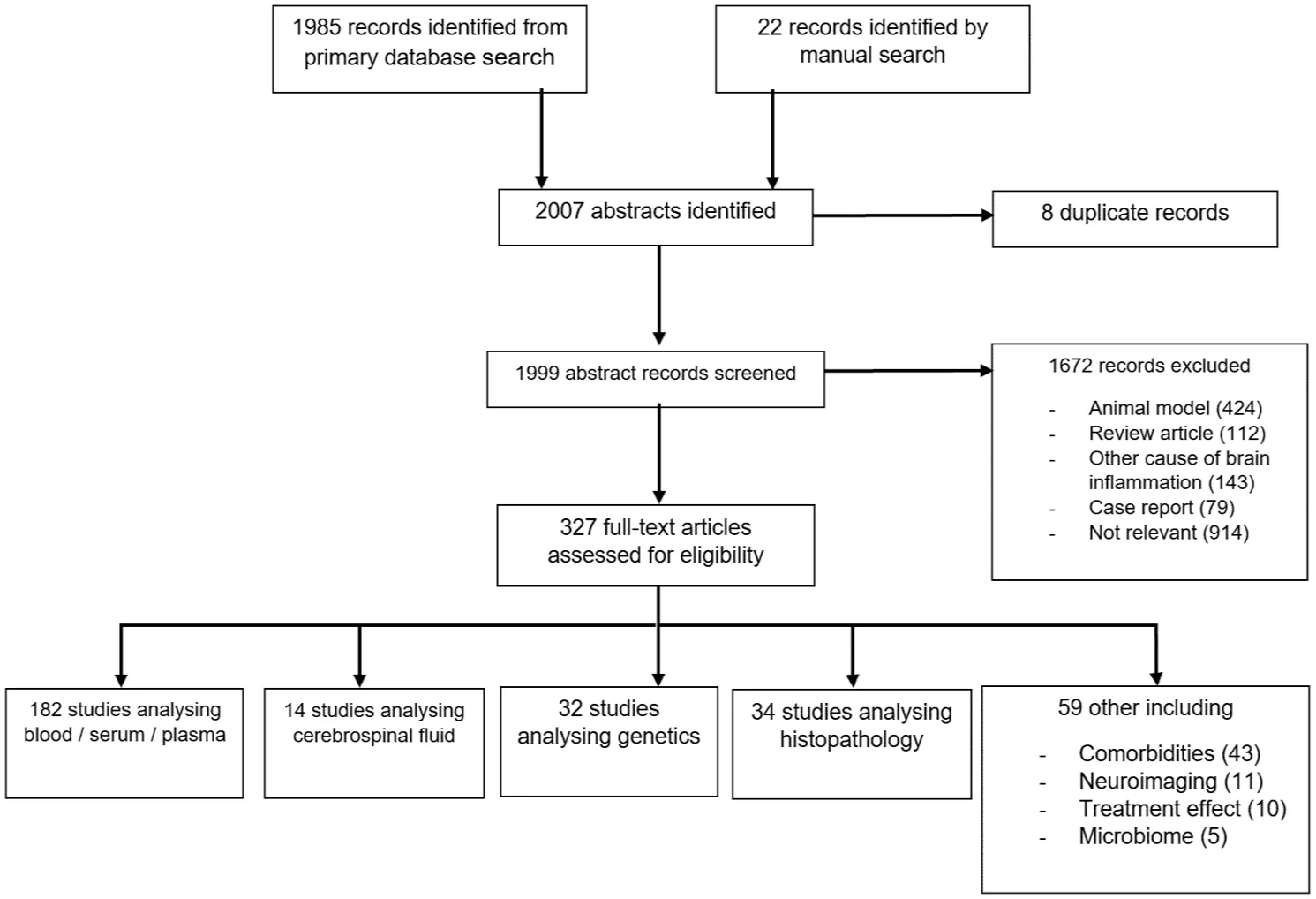
PRISMA flowchart showing literature search and selection of studies. Note that some studies were categorized as assessing inflammation in multiple domains. PRISMA: Preferred Reporting Items for Systematic Reviews and Meta-Analysis.

## Narrative review

### Insights from preclinical studies

Although preclinical studies using animal models are not the focus of this review, evidence of a possible role of inflammation in SVD/VCID comes from several models of cerebral hypoperfusion, including surgical occlusion or stenosis of one or both carotid arteries or the middle cerebral artery (usually in rodents). These models recapitulate some (but not all) aspects of SVD and VCID.^20,21^ Models for cardiovascular risk factor-mediated SVD include the spontaneously hypertensive/stroke-prone rat model (SHRSP)^22^ and familial forms of SVD such as cerebral autosomal dominant arteriopathy with subcortical infarcts and leukoencephalopathy (CADASIL)^23^ and CAA.^24^ These models show changes in brain leucocyte populations and microglial morphology,^22^ increased intracellular signaling molecules such as hypoxia-inducible factor-1α,^25^ extracellular matrix (ECM) metalloprotease activity,^25^ blood–brain barrier (BBB) disruption,^26^ and brain endothelium dysfunction.^27^

These neuroinflammatory processes can be targeted therapeutically in preclinical models. For example, overexpression of triggering receptor expressed on myeloid cells-2 (TREM2), an anti-inflammatory microglial receptor, was associated with a reduction in inflammatory cytokines and improved spatial learning in mice with bilateral carotid artery occlusion.^28^ Treatment with minocycline, an anti-inflammatory antibiotic that inhibits matrix metalloprotease (MMP) activity, led to reduced inflammatory markers, less white matter damage, and improved behavior in a SHRSP model,^25^ while inhibition of colony-stimulating factor-1 receptor (CSF1R), a receptor that regulates microglial proliferation, attenuated white matter damage in a bilateral carotid stenosis mouse model.^29^ These studies highlight a few relevant pathways that might be targeted in patients.

### Central nervous system inflammation and SVD/VCID in humans

#### Neuropathological studies

Histopathological studies in sporadic SVD show evidence of activated microglia around WMH lesions, particularly in patients with cardiovascular risk factors such as hypertension.^30^ Furthermore, activated microglia are associated with radiological markers of SVD and are particularly prevalent in the subventricular areas.^31^ Immunohistochemical analysis has revealed increased immunoreactivity of thrombomodulin, a component of the endothelial-derived coagulation cascade,^32^ and glial fibrillary acidic protein (GFAP), an astrocytic structural protein^31^ in the brains of patients with both symptomatic sporadic SVD and CADASIL.

Analysis of post-mortem samples from patients with lobar intracerebral hemorrhage (ICH) resulting from CAA shows macrophages associated with vascular β-amyloid deposition and microglia co-localized with β-amyloid in brain parenchyma.^33^ Monocyte/macrophage infiltration also occurs in genetic forms of CAA,^34^ as does lymphocytic infiltration from the vascular compartment.^35^ More recently, *ex vivo* magnetic resonance imaging (MRI) scans with parallel histological analysis show the presence of activated microglia and BBB leakage in arterioles most severely affected by CAA.^36^

Post-mortem transcriptomic analysis has been applied in vascular dementia, with single-nucleus transcriptomic data showing upregulated cytokine signaling in microglia and endothelial activation.^37^ Although there are limitations in interpreting the timing of these changes, taken together, these studies suggest that both vascular inflammation and innate immune cell activity may be relevant in SVD/VCID.

#### Cerebrospinal fluid analysis

CSF sampling offers a more direct way of measuring immune/inflammatory changes in the CNS compartment than blood tests. Several proteins related to inflammation have been identified in CSF in patients with SVD^38^ and VCID,^39^ including interleukin (IL)-6, and CD14, a microglial surface marker, which predicted conversion from mild cognitive impairment to vascular dementia.^40^ Other markers of potential relevance include procalcitonin, a component of the systemic inflammatory response, which was higher in the CSF but not the blood of patients with VCID compared with controls, implying CNS production.^41^

Proteomic data from the CSF of patients with SVD/VCID are lacking, but one study using a multiplex immunoassay investigated 13 markers of inflammation, endothelial injury, and angiogenesis in the CSF of patients with mild cognitive impairment and found an association between WMH volume and several candidate markers, including IL-6 and VEGF.^42^ Cytometric analysis of CSF (containing mainly T-lymphocytes) indicates that patients with vascular dementia have a significantly lower proportion of CD4+ memory T cells,^43^ implying a failure of regulation of neuroinflammation within the CNS. These cells may transit between the systemic circulation and the brain,^44^ providing some evidence of communication between the two compartments in VCID.

#### Neuroimaging of inflammation in humans

Imaging studies can also provide insight into brain inflammation. Positron emission tomography (PET) is most frequently used with radioligands targeted against the translocator surface protein (TSPO) that is strongly expressed in activated microglia (sample images showing focal areas of increased TSPO signal are shown in Figure 3). Increased TSPO PET signal was reported in both WMH and “normal-appearing white matter” of patients with severe SVD,^45^ and the TSPO signal in specific brain regions is better correlated with radiological features of sporadic SVD than to CAA.^46^ This indicates that microglial activation can be detected in SVD, but does not tell us whether it is causal or merely secondary to tissue damage. Longitudinal analysis has shown lower TSPO signal in brain areas that become new WMH over 1 year,^47^ suggesting that microglial activation may occur sometime before tissue becomes visibly damaged, or that the association is not causal. Further longitudinal studies are required to determine whether increased TSPO binding predicts clinicoradiological progression.

**Figure 3.**
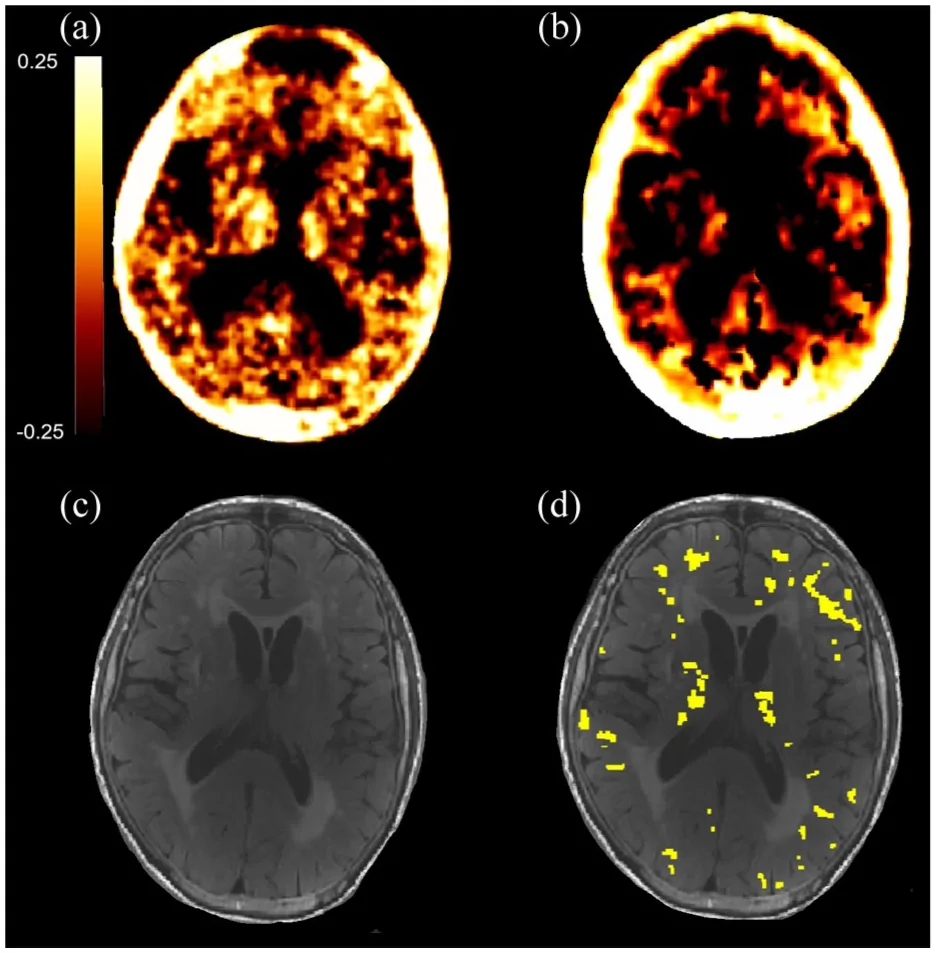
Representative PET images from (a) a patient with sporadic SVD and (b) an age-matched healthy control participant, acquired after injection of ^11^C-PK11195, with binding potential (relative to reference tissue) indicated by the color bar showing increased binding in white matter and deep gray matter in the patient images. Note that binding potential is significantly higher in bone, and the skull is removed for analysis. (c) Corresponding fluid attenuated inversion recover (FLAIR) image for the patient in (a). (d) “Hotspots” of increased ^11^C-PK11195 binding (greater than the 95th percentile of reference tissue in control participants) are shown overlaid on the FLAIR image from the patient.

### Systemic inflammation and SVD/VCID in humans

#### Specific candidate biomarkers from blood

The association between specific inflammatory proteins has been reviewed in detail in sporadic SVD and CAA^18^ and in VCID of any etiology.^48^ Molecules of interest include proteins involved in coagulation, endothelial cell activation, immune cell adhesion, and cytokines forming part of systemic inflammatory pathways.

Fibrinogen levels were found to be associated with markers of SVD,^49^ WMH progression, incident vascular dementia,^50^ and endothelial dysfunction. Homocysteine, an amino acid associated with endothelial dysfunction and thrombosis, is associated with several markers of SVD.^51,52^ Intracellular adhesion molecule (ICAM)-1 is associated with WMH and silent infarcts in patients with ischemic stroke^53^ and population-based cohorts,^54^ and levels predict future WMH progression.^53^ Other adhesion molecules, including P-selectin,^55^ E-selectin, and vascular cell adhesion molecule (VCAM)-1,^56^ correlate with SVD markers such as asymptomatic lacunes and WMHs, underlining the importance of endothelial activation.

Proteins from canonical systemic inflammatory pathways, such as C-reactive protein (CRP), predict WMH burden and progression, though not diffusion tensor imaging parameters, which may be a more sensitive measure of white matter microstructural damage.^57^ Similar associations are seen with IL-6,^58,59^ which drives CRP production and predicts functional impairment in VCID.^60^ Several reviews and meta-analyses have shown that levels of these key inflammatory molecules correlated with the risk of VCID.^48,61,62^

However, the extent to which these markers are causally related remains uncertain. Mendelian randomization experiments in which genetic variants are used as a proxy for an exposure of interest have been used to assess causality. Genetically determined levels of several markers of endothelial activation were associated with cognitive impairment and SVD markers on MRI, for example platelet endothelial aggregation factor receptor-1.^63^ Further studies show a bidirectional relationship between increased total lymphocyte count and multiple subtypes of vascular dementia,^64^ and associations between several genes that encode immune proteins and the risk of developing vascular dementia, including positive correlations with chemokines such as C-X-C Motif Chemokine Ligand 11 (CXCL11)^65^ and cytokines such as macrophage inflammatory protein 1b (MIP-1b).^66^ These findings suggest that both innate and adaptive immune cells modulate the risk of SVD/VCID.

Table 1 summarizes the associations of inflammatory markers that have emerged from Mendelian randomization studies, and Supplementary Table 1 details the associations found between specific biomarkers and both radiological markers of SVD severity and clinical diagnosis of VCID.

**Table 1.** Associations between genetically determined levels of inflammatory markers in blood and diagnosis or clinicoradiological markers of severity of SVD/VCID in Mendelian randomization studies.

Reference	Instrument representing inflammation	Instrument representing VCID/SVD severity	Findings
Han et al.^67^	GWAS summary data for a panel of 91 cytokines (European Bioinformatics Institute GWAS database)	GWAS summary data for WMH volume, diffusion tensor imaging parameters, cerebral microbleeds, and lacunar stroke (Cerebrovascular Disease Knowledge Portal database)	WMH volume correlated with delta/Notch-like EGF repeat containing protein, fibroblast growth factor-19, and monocyte chemoattractant protein-3; WMH volume inversely correlated with IL-1-α, TGF-α, and macrophage inflammatory protein-1
Tchuisseu-Kwangoua et al.^68^	GWAS data for 26 variants near IL-6 receptor and an IL-6 receptor missense variant (CHARGE consortium and UK Biobank)	GWAS summary data for small vessel stroke, vascular dementia, and non-lobar ICH (multiple pooled international cohorts)	IL-6 is associated with carotid plaque and risk of large artery stroke but not risk of small vessel stroke, vascular dementia, or non-lobar ICH
Sun et al.^63^	GWAS summary data for a panel of 996 cytokines and endothelial proteins (UK Biobank and Iceland 36K cohorts)	GWAS summary data for small vessel stroke, WMH volume, diffusion tensor imaging, PVS burden, and CMBs (pooled analysis of multiple pooled international cohorts)	12 proteins associated with multiple neuroimaging markers of SVD: APOE, CD46, EPHA2, FTL4, HEXIM1, MEGF10, MERTK, METAP1D, NPTXX1, PDE5A, PEAR1, TIMD4
Liu et al.^64^	GWAS summary data for lymphocyte count (UK Biobank; Blood Cell Consortium)	GWAS summary data for five subtypes of vascular dementia (FinnGen)	Vascular dementia risk across multiple phenotypes (subcortical dementia, dementia due to multiple infarcts, sudden onset vascular dementia, mixed dementia, and otherwise undefined vascular dementia) correlated with lymphocyte count
Li et al.^69^	GWAS summary data for a panel of 91 cytokines (multiple pooled international cohorts)	GWAS summary data for WMH volume, diffusion tensor imaging, CMBs, PVS burden, and lacunar stroke (UK Biobank)	WMH volume correlated with eukaryotic translation initiation factor 4E-binding protein-1 PVS inversely correlated with natural killer cell receptor 2B4 Small vessel stroke risk correlated with C-C motif chemokine 25, TNF-related apoptosis-inducing ligand, and TNF ligand superfamily member 12
Yang Y et al.^70^	GWAS summary data for a panel for 41 cytokines (Young Finns Study, FINRISK1997 and FINRISK2002)	GWAS summary data for WMH volume and diffusion tensor imaging parameters (CHARGE consortium and UK Biobank)	WMH volume and mean diffusivity correlated with IL-18 levels Fractional anisotropy inversely correlated with IL-18 and MCP-1
Qiao et al.^71^	GWAS summary data for a panel of 24 cytokines and cell adhesion molecules (IEU and EBI databases)	GWAS summary data for WMH volume, mean diffusivity, and PVS (UK Biobank)	Increased small vessel stroke risk correlated with TNF-related apoptosis-inducing ligand and IL-1 receptor-like 2 levels Increased PVS burden correlated with E-selectin, IL-3 receptor subunit α, and IL-5 receptor subunit α levels

APOE: apolipoprotein E; CD46: membrane cofactor protein; EPHA2: ephrin type-A receptor 2; FLT4: vascular endothelial growth factor receptor 3; GWAS: genome-wide association study; HEXIM1: hexamethylene bis-acetamide-inducible protein 1; ICH: intracerebral hemorrhage; IL: interleukin; MCP-1: monocyte chemoattractant protein 1; MEGF10: multiple epidermal growth factor-like domains protein 10; MERTK: tyrosine-protein kinase Mer; METAP1D: methionine aminopeptidase 1D, mitochondrial; NPTX1: neuronal pentraxin-1; PDE5A: cGMP-specific 3',5'-cyclic phosphodiesterase; PEAR1: platelet endothelial aggregation receptor 1; PVS: perivascular spaces; TGF-α: tumor growth factor α; TIMD4: T-cell immunoglobulin and mucin domain–containing protein 4; TNF: tumor necrosis factor; WMH: white matter hyperintensity lesions.

#### Proteomic analysis

Proteomics platforms using multiplex assays allow simultaneous measurement of large numbers of proteins. In patients with SVD, a compound biomarker score derived from a panel of cardiovascular inflammation biomarkers correlated with WMH volume, lacune count, CMB count, and PVS burden.^72^ The cluster included cytokines such as members of the tumor necrosis factor α (TNFα) receptor superfamily, platelet activation markers, and coagulation proteins such as von Willebrand factor. This panel has been measured in patients with severe SVD, and the first principal component of the data correlated significantly with BBB leakage measured using contrast-enhanced MRI.^45^ Similar analysis has been performed in patients with CADASIL, showing evidence of increased levels of several pro-inflammatory molecules, including monocyte chemoattractant protein-1 (MCP-1), IL-8, and fibroblast growth factor-19.^73,74^

In a memory clinic cohort diagnosed with vascular dementia, levels of two macrophage-related chemokines were significantly higher compared with controls;^75^ another study found that in a population-based cohort, several chemokines correlated with performance in multiple cognitive domains and overall cognitive performance, including C-C motif chemokine ligand 11 (CCL11) and C-X-C motif chemokine ligand 9 (CXCL9).^76^ A compound inflammatory biomarker score derived from the UK Biobank cohort was related to cognitive performance and predicted vascular dementia.^77^

#### Analysis of peripheral blood cytology

Systemic inflammation can be estimated using the neutrophil-to-lymphocyte ratio (NLR) and systemic immune-inflammatory index (SII = platelet count × neutrophil count / lymphocyte count) derived from routine cell counts measured in clinical care. Increases in these indices are proxy measures of inflammation and are associated with radiological signs of SVD^78,79^ and predict dementia,^80^ although they may correlate more specifically with cardiovascular risk factors.^81^

#### Immunophenotyping—peripheral blood

More detailed assessment of immune cell phenotype and activity can be performed using flow cytometry or RNA sequencing, or by measuring the functional response of immune cells. This includes not only cells of the adaptive immune system (mainly lymphocytes), which may mediate the increased cardiovascular risk seen in autoimmune disease,^82^ but also innate immune cells, including monocytes and macrophages. “Trained” innate immune cells showing an enhanced response to repeated stimulation may underlie some of the chronic pro-inflammatory changes driven by atherosclerosis^83^ and could be relevant in SVD.

Immunophenotyping studies have provided evidence for alterations in circulating cell populations in VCID; flow cytometry showed fewer T and B lymphocytes mediated by a reduction in FoxP3+ regulatory T cells that have anti-inflammatory activity.^84^ Reduced regulatory T-cell activity is a consistent finding in VCID and correlates with executive dysfunction^85^ as well as dementia progression.^86^ Immune cell activation can also be tested by measuring pro-inflammatory secreted molecules, including neutrophil extracellular traps, large circulating structures of DNA, and granulocytic enzymes that are found in higher concentration in patients with SVD and predict cognitive impairment.^87^ A shift toward pro-inflammatory subpopulations is seen in peripheral monocytes in patients with SVD,^88^ and the cytokine production capacity of these cells is increased and predicts WMH progression.^89^

### Genetic predisposition to inflammation in SVD/VCID

There is evidence that genetic variants in proteins from canonical systemic inflammatory pathways, such as IL-6 and CRP, are associated with an increased burden of SVD,^90^ but subsequent larger genome-wide association studies have not replicated these results, finding instead that genes related to microglial activation,^91^ ECM degradation, and cell adhesion molecules^92^ appear most relevant in SVD. Genetically downregulated IL-6 (taken as a proxy for therapeutic IL-6 blockade) showed no association with imaging markers of SVD or cognition.^93^

### Systemic inflammation related to comorbidities

Several studies have investigated the relationship between SVD markers or cognitive impairment and other comorbid health conditions, particularly those with an inflammatory basis, such as infectious or autoimmune diseases. Imaging markers of SVD are more prevalent in rheumatoid arthritis,^94^ systemic lupus erythematosus,^95^ and inflammatory bowel disease,^96^ as well as metabolic or cardiovascular conditions such as non-alcoholic fatty liver disease and carotid atherosclerosis.

Similar associations were found in infection, with evidence suggesting that vascular cognitive impairment develops more frequently in diabetic patients after any infectious disease requiring hospital admission^97^ and radiological signs of SVD are more prevalent in specific infections such as *Helicobacter pylori*.^98^

### Role of the gut microbiome

It has been hypothesized that gastrointestinal bacteria can affect the risk of cardiovascular and neurological conditions by inducing a low-grade systemic inflammatory response.^99^ This has been investigated in SVD in several small studies, providing evidence that gut microbiota from patients with SVD are more immunogenic^100^ and that altered bacterial composition is associated with changes in cerebral blood flow and cognitive performance in VCID.^101^ Genetic association studies suggest that there may be a causative association between certain bacterial species in the microbiome and both circulating cytokines and the diagnosis of vascular dementia.^102^

### Can targeting inflammation treat SVD and VCID?

Few clinical trials have tested interventions to reduce inflammation in SVD and VCID. A summary of completed and ongoing trials or those testing outcomes in these subgroups is shown in Table 2; this excludes trials of treatment in the acute phase of stroke in which the treatment period was not long enough to modify chronic disease processes. The most frequently tested drugs are minocycline and colchicine, which have rather non-specific anti-inflammatory properties.

**Table 2.** Completed and ongoing clinical trials of anti-inflammatory medication in the chronic phases of SVD/VCID.

Study	Status	Study design	Population	Intervention	Control	Primary outcome	Results
Primary studies of patients with SVD/VCID							
MINERVA^103^	Completed	Single-center randomized, double-blind trial	Severe and symptomatic sporadic SVD (*n* = 44)	Minocycline—3 months	Placebo	“Hotspots” of increased microglial signal on ^11^C-PK11195 PET	Neutral
Bax et al.^104^	Completed	Single-center case series	Severe CAA with recurrent ICH (*n* = 16)	Minocycline—median 12.4 months	N/A	ICH incidence rate in the year post-treatment compared with the year pre-treatment	Significant reduction in ICH rate
Jia et al.^105^	Completed	Single-center randomized, double-blind trial	Patients with VCI but not dementia (*n* = 281)	DL-3-n-butylphthalide—6 months	Placebo	Change in Alzheimer’s disease assessment scale—cognitive subscale score	Significantly greater improvement in the Butylphthalide group
BATMAN^106^	In progress	Single-center randomized, double-blind trial	Monogenic CAA (*n* = 30) and sporadic CAA (*n* = 30)	Minocycline—3 months	Placebo	Cerebrospinal fluid biomarkers of inflammation	*Pending*
CARE-ICH^107^	In progress	Multicenter randomized double-blind trial	CAA with ICH and CSS or ⩾2 ICHs	Colchicine—12 months	Placebo	Adverse events and tolerability	*Pending*
Secondary analyses of larger studies, including SVD/VCID as a subgroup							
CONVINCE^108^	Completed	Multicenter randomized open-label blinded endpoint trial	Unselected patients with minor stroke or high-risk TIA (*n* = 3154; stroke due to SVD *n* = 854)	Colchicine—24 months	N/A	Recurrent major cardiovascular event, including stroke	Neutral

CSS: cortical superficial siderosis; ICH: intracerebral hemorrhage; TIA: transient ischemic attack.

Minocycline, which has several potentially therapeutic effects including inhibition of MMPs and BBB stabilization,^109^ reduced white matter damage and improved functional outcomes in animal models.^25,110^ However, in a phase 2 randomized controlled trial (RCT), a 3-month treatment course had no effect on the TSPO ligand ^11^C-PK11195 signal measured using PET.^103^ Minocycline has been suggested as a treatment option for CAA, supported by animal models,^111^ and one single-center report found a significant ICH reduction when prescribed off-label to patients with aggressive CAA.^104^ This is being further tested in patients with both sporadic and familial CAA.^106^

Colchicine is an anti-inflammatory medication that disrupts microtubule formation, thereby inhibiting the mitosis of immune cells and inflammasome formation. It suppresses macrophage and monocyte activation in response to atherosclerosis.^112^ Subgroup analysis of the large CONVINCE trial in patients with non-cardioembolic stroke found that in those with small vessel stroke, there was a small and statistically nonsignificant reduction in major adverse cardiovascular events.^108^ Further studies are required to confirm this finding and assess whether there is any benefit on cognition.

Future trials of compounds inhibiting specific immune/inflammatory pathways are, therefore, urgently required to confirm any causal contribution of inflammation to SVD and VCID.

## Discussion

We reviewed studies using a variety of methodologies to demonstrate that both peripheral and central inflammation occur in SVD/VCID. However, the strength of the association is heterogeneous, and it remains to be determined if these associations are causal and whether interventions targeting inflammation can alter disease progression.

Preclinical models are limited by several factors, including the lack of a truly representative model of human SVD/VCID, and fundamental differences between human and non-human immune systems,^113^ but provide several mechanistic pathways that can be tested in patients. Key pathways include endothelial cell dysfunction, activation of innate immune cells, including circulating monocytes and microglia within the CNS, BBB breakdown, and degradation of the ECM. Many of these processes have also been demonstrated in patients, including endothelial activation,^114,115^ pro-inflammatory changes in innate immune cells,^88^ and microglial activation.^45^ Figure 4 shows a hypothesized mechanism for the interaction between these processes.

**Figure 4.**
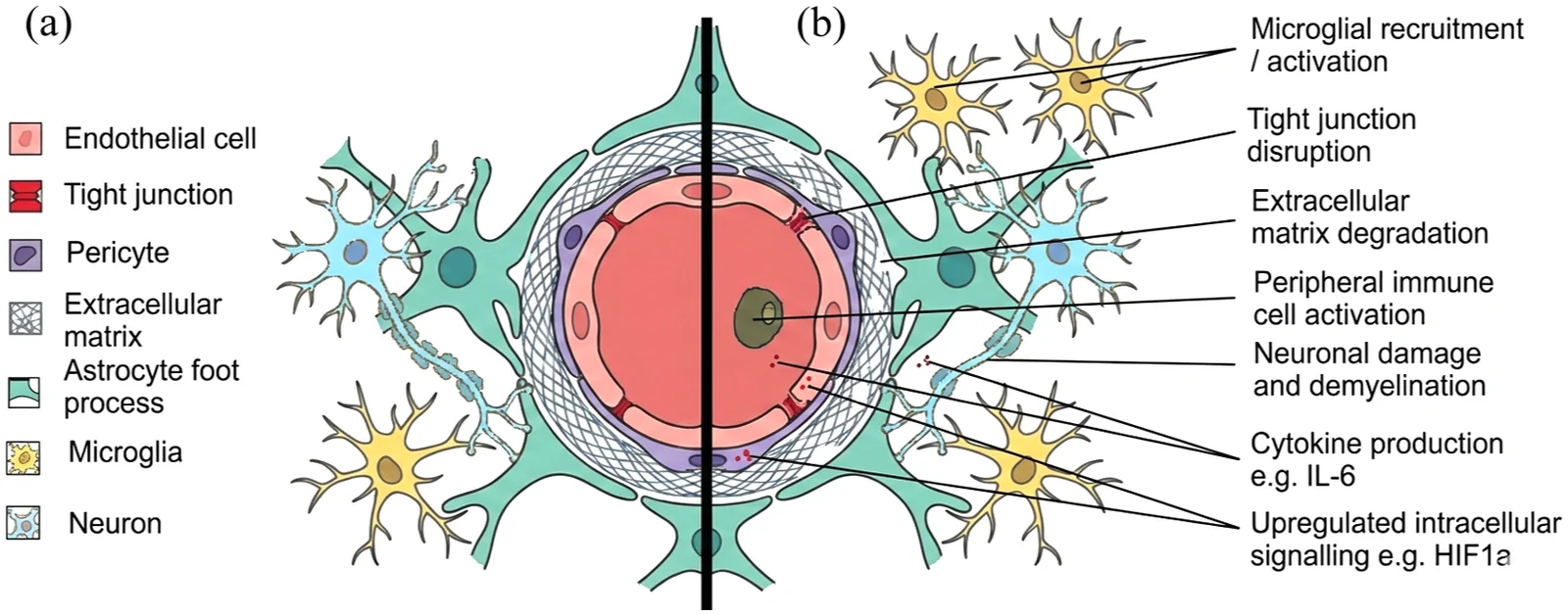
(a) Structure of the healthy blood–brain barrier composed of endothelial cells, pericytes, astrocytes, and extracellular matrix, and its relationship to neuroglial cells and (b) pathogenic mechanisms causing inflammation and disruption of the blood–brain barrier including upregulation of intracellular signaling molecules, release of pro-inflammatory cytokines both in the systemic circulation and in brain tissue, degradation of the extracellular matrix, disruption of endothelial tight junctions, and recruitment of microglial cells.

Most candidate biomarkers of inflammation have been assessed in blood, as this is more practical and acceptable than CSF sampling. These have produced some varying results about which pathways are most closely associated with disease (see Supplementary Table 1); the most consistent results have come from investigation of pro-inflammatory cytokines, including IL-6, an acute phase response protein that broadly stimulates innate immune cells and can cross the BBB.^116^ Meta-analysis, including some of the studies we identified, confirms significantly raised serum IL-6 levels in patients with VCID compared with controls and that this predicts incident vascular dementia.^48^ These results suggest that suppression of the acute phase response, including innate immune cells, may be a potential therapeutic option.

Whether immune activation, either systemically or in the CNS, is directly causative for disease progression is uncertain. Longitudinal studies suggest that inflammation predicts clinical or radiological disease progression, but to answer this question fully, it will be necessary for successful clinical trials to demonstrate not just that the pathophysiological process can be reversed, but that this leads to a corresponding improvement in disease outcome. Systemic inflammation might reduce brain resilience to ischemia or arise as a direct response to CNS tissue damage.^11,19,56^

The timeline of inflammatory changes is also uncertain. It may be that by the time tissue damage reaches a particular threshold, inflammatory processes are no longer driving disease and, therefore, any intervention would have to be delivered before this. Many of the studies summarized above are limited by assessment at a single time point, so we cannot be certain when immune cell changes occur. Genetic studies have similar limitations as they test lifetime risk scores. Repeatability of many of the biomarkers summarized above is limited by tolerability (e.g., CSF sampling) or radiation exposure (PET imaging). Blood biomarkers from ongoing large multi-site consortia^117^ may provide the best opportunity to obtain repeat samples that reveal how immune processes develop over time.

Early data on the effectiveness of anti-inflammatory treatment in SVD/VCID have not shown a clear benefit, but few studies have been performed. Given the range of different inflammatory mechanisms that have been elucidated, it may be necessary to target multiple pathways, for example, blockade of systemic inflammatory pathways together with small molecules that inhibit MMPs within the CNS or induce anti-inflammatory microglial phenotypes. These potential treatments are currently limited to preclinical use. Finally, it is hoped that a better understanding of which individual components of the inflammatory pathways are causally related to SVD/VCID risk will allow for more targeted therapies.

## Supplemental Material

sj-docx-1-wso-10.1177_17474930261461366 – Supplemental material for The role of inflammation in cerebral small vessel disease and vascular cognitive impairment, and therapeutic implications Supplemental material, sj-docx-1-wso-10.1177_17474930261461366 for The role of inflammation in cerebral small vessel disease and vascular cognitive impairment, and therapeutic implications by Robin B Brown, Stuart M Allan and Hugh S Markus in International Journal of Stroke
