# Supplementary material for: The role of inflammation in cerebral small vessel disease and vascular cognitive impairment, and therapeutic implications
Source: Int J Stroke. 2026 Jun 8;21(9):1246–59. doi: 10.1177/17474930261461366 (PMC13672893; doi:10.1177/17474930261461366)
Supplement: Supplementary file 1 [file sj-docx-1-wso-10.1177_17474930261461366.docx]

**SUPPLEMENTARY MATERIAL**

**The role of inflammation in cerebral small vessel disease and vascular cognitive impairment, and therapeutic implications**

Robin B. Brown^1*^, Stuart M. Allan^2,3^, Hugh S. Markus^1^

^1^Department of Clinical Neurosciences, University of Cambridge, Cambridge, UK

^2^Division of Neuroscience, School of Biological Sciences, Faculty of Biology, Medicine and Health, University of Manchester, Manchester, UK

^3^Geoffrey Jefferson Brain Research Centre, Manchester Academic Health Science Centre, Northern Care Alliance NHS Foundation Trust, University of Manchester, Manchester, UK

*Corresponding author

Email: rbb29@cam.ac.uk

Telephone: +44 01223 256661

X: @rbb_29, @CamStroke

**Table S1. Association between blood biomarkers and SVD / VCID**

Table S1 shows the associations between inflammatory markers measured in blood, and either markers of SVD severity (WMHs, white matter hyperintensities; CMBs, cerebral microbleeds; PVSs, enlarged perivascular spaces) or diagnosis of VCID

| Biomarker | Positive correlations | Neutral / no correlation | Negative correlations |
| --- | --- | --- | --- |
| Clotting cascade | | | |
| d-dimer | Gupta et al., 2005 (VaD)^1^  Hao et al., 2024 (Lacunar stroke)^2^ | Gallacher et al., 2010 (VaD)^3^  Wiseman et al., 2015 (Lacunar stroke)^4^ |  |
| Fibrinogen | Aono et al., 2006 (WMH)^5^  Gallacher et al., 2010 (VaD)^3^  Gao et al., 2023 (SVD)^6^  Marti-Fabregas et al., 2002 (WMHs)^7^  Zhang et al., 2022 (SVD score)^8^ | Gupta et al., 2005 (VaD)^1^  Wiseman et al., 2015 (Lacunar stroke)^4^ |  |
| Tissue plasminogen activator | Gallacher et al., 2010 (VaD)^3^ | Knottnerus et al., 2010 (WMHs, lacunes)^9^  Wiseman et al., 2015 (Lacunar stroke)^4^ | Wiseman et al., 2015 (Lacunar stroke)^4^ |
| Plasminogen activator inhibitor | Gupta et al., 2005 (VaD)^1^  Knottnerus et al., 2010 (WMHs, lacunes)^9^ |  |  |
| Von Willebrand Factor | Gupta et al., 2005 (VaD)^1^  Kearney-Schwartz et al., 2009 (WMHs)^10^ | Gallacher et al., 2010 (VaD)^3^  Knottnerus et al., 2010 (WMHs, lacunes)^9^ |  |
| Vascular inflammation and endothelial activation markers | | | |
| Homocysteine | Cao et al., 2021 (Lacunes, WMHs)^11^  Chen et al., 2024 (WMH – women only)^12^  Dufouil et al., 2003 (cognition)^13^  Hassan et al., 2004 (Lacunes, WMHs)^14^  Hogervorst et al., 2002 (WMHs)^15^  Kloppenborg et al., 2014 (WMHs, lacunes)^16^  Longstreth et al., 2004 (Combined SVD markers)^17^  Naka et al., 2006 (WMHs)^18^  Narayan et al., 2011 (atrophy)^19^  Pavlovic et al., 2011 (WMHs)^20^  Raz et al., 2012 (WMHs)^21^  Staszewski et al., 2018 (SVD progression)^22^  Vermeer et al., 2002 (WMHs, lacunes)^23^  Zhang DD et al., 2022 (Lacunes)^24^ | Gunstad et al., 2006 (VCI, WMH)^25^  Longstreth et al., 2004 (WMH, lacunes)^17^  Naka et al., 2006 (CMBs)^18^  Narayan et al., 2011 (WMHs, lacunes)^19^  Zhang L et al., 2022 (SVD score)^8^  Zhang DD et al., 2022 (WMHs, PVS, CMBs)^24^ |  |
| ICAM-1 | Engelhardt et al., 2004 (VaD)^26^  Gregory et al., 2019 (VCI)^25^  Markus et al., 2005 (WMHs)^27^ | Staszewski et al., 2018 (SVD progression)^22^  Zhang DD et al., 2022 (WMHs, Lacunes, PVS, CMBs)^24^  Wiseman et al., 2015 (Lacunar stroke)^4^ |  |
| Thrombomodulin |  | Knottnerus et al., 2010 (WMHs, lacunes)^9^ |  |
| VCAM-1 | Zhang DD et al., 2022 (WMHs)^24^ | Elahi et al., 2018 (VaD)^28^  Engelhardt et al., 2004 (VaD)^26^  Zhang DD et al., 2022 (Lacunes, PVS, CMBs)^24^ |  |
| E-selectin |  | Zhang DD et al., 2022 (WMHs, Lacunes, PVS, CMBs)^24^ |  |
| P-selectin |  | Zhang DD et al., 2022 (WMHs, Lacunes, PVS, CMBs)^24^  Staszewski et al., 2018 (SVD progression)^22^ |  |
| Cytokines | | | |
| CRP | Cao et al., 2021 (Lacunes, WMHs)^11^  Engelhardt et al., 2004 (VaD)^26^  Gunstad et al., 2006 (VCI)^25^  Gupta et al., 2005 (VaD)^1^  Hilal et al., 2016 (WMH, lacunes, PVSs, CMBs)^29^  Hoshi et al., 2006 (Covert infarct)^30^  Hsu et al., 2017 (VaD)^31^  Mitaki et al., 2016 (Lacunes)^32^  Raz et al., 2012 (WMHs)^21^  Yao et al., 2019 (WMHs)^33^  Zhang et al., 2022 (SVD score)^8^ | Chen et al., 2024 (WMH)^12^  Gallacher et al., 2010 (VaD)^3^  Gunstad et al., 2006 (WMH)^25^  Helmy et al., 2012 (VaD)^34^  Huang et al., 2022 (WMHs)^35^  Mitaki et al., 2016 (WMHs, CMBs)^32^  Miwa et al., 2016 (VaD)^36^  Ohara et al., 2019 (VaD)^37^  Satizabal et al., 2013 (PVSs)^38^  Staszewski et al., 2018 (SVD progression)^22^  Zhang DD et al., 2022 (WMHs, Lacunes, PVS, CMBs)^24^  Wada et al., 2008 (WMHs)^39^  Wiseman et al., 2015 (Lacunar stroke)^4^ |  |
| IFN-γ | Bai et al., 2025 (CADASIL)^40^ | Cai et al., 2025(VaD)^41^ |  |
| TNF-α | Bai et al., 2025 (CADASIL)^40^  Cai et al., 2025(VaD)^41^  Jefferson et al., 2007 (brain atrophy)^42^  Zuliani et al., 2007b (VaD)^43^ | Hanning et al., 2016 (WMHs)^44^  Staszewski et al., 2018 (SVD progression)^22^  Zuliani et al., 2007a (VaD)^45^  Zhang DD et al., 2022 (WMHs, Lacunes, PVS, CMBs)^24^  Wiseman et al., 2015 (Lacunar stroke)^4^ |  |
| TNF-β | Bai et al., 2025 (CADASIL)^40^ |  |  |
| IL-1β | Bai et al., 2025 (CADASIL)^40^ Cai et al., 2025 (VaD)^41^  Zuliani et al., 2007b (VaD)^43^ | Hanning et al., 2016 (WMHs)^44^  Zuliani et al., 2007a (VaD)^45^ |  |
| IL-2 |  | Bai et al., 2025 (CADASIL)^40^  Cai et al., 2025(VaD)^41^ |  |
| IL-4 | Bai et al., 2025 (CADASIL)^40^ | Cai et al., 2025(VaD)^41^ |  |
| IL-5 | Bai et al., 2025 (CADASIL)^40^ | Cai et al., 2025(VaD)^41^ |  |
| IL-6 | Bai et al., 2025 (CADASIL)^40^ Cai et al., 2025 (VaD)^41^  Dukic et al., 2016 (VaD)^46^  Gupta et al., 2005 (VaD)^1^  Hanning et al., 2016 (WMH)^44^  Helmy et al., 2012 (VaD)^34^  Hoshi et al., 2006 (Covert infarct)^30^  Jefferson et al., 2007 (brain atrophy)^42^  Liu et al., 2024 (VCI)^47^  Miwa et al., 2016 (VaD)^36^  Satizabal et al., 2013 (PVSs)^38^  Staszewski et al., 2018 (SVD progression)^22^  Zuliani et al., 2007a (VaD)^45^  Zuliani et al., 2007b (VaD)^43^ | Elahi et al., 2018 (VaD)^28^  Gallacher et al., 2010 (VaD)^3^  Oberheiden et al., 2010 (WMHs)^48^  Zhang DD et al., 2022 (WMHs, Lacunes, PVS, CMBs)^24^ |  |
| IL-7 |  |  | Oberheiden et al., 2010 (WMHs)^48^ |
| IL-8 | Bai et al., 2025 (CADASIL)^40^  Hanning et al., 2016 (WMH)^44^ | Cai et al., 2025(VaD)^41^  Zuliani et al., 2007 (VaD)^45^ |  |
| IL-10 |  | Bai et al., 2025 (CADASIL)^40^  Cai et al., 2025(VaD)^41^  Hanning et al., 2016 (WMH)^44^  Zuliani et al., 2007b (VaD)^43^ |  |
| IL-12p70 |  | Cai et al., 2025(VaD)^41^  Hanning et al., 2016 (WMH)^44^ |  |
| IL-17F | Bai et al., 2025 (CADASIL)^40^ |  |  |
| IL-18 | Altendahl et al., 2020 (WMH)^49^  Malaguarnera et al., 2006 (VaD)^50^ |  |  |
| IL-22 | Bai et al., 2025 (CADASIL)^40^ |  |  |
| CXCL4 | Karel et al., 2022 (WMHs)^51^ |  |  |
| CXCL7 | Karel et al., 2022 (WMHs)^51^ |  |  |
| TGFβ | Malaguarnera et al., 2006 (VaD)^50^ | Zuliani et al., 2007 (VaD)^45^ |  |
| Chemoattractants and co-stimulatory molecules | | | |
| CTLA-4 |  | Busse et al., 2002 (VaD)^52^ |  |
| CD28 |  | Busse et al., 2002 (VaD)^52^ |  |
| CD40L | Staszewski et al., 2018 (SVD progression)^22^ | Oberheiden et al., 2010 (WMHs)^48^  Zhang DD et al., 2022 (WMHs, Lacunes, PVS, CMBs)^24^ |  |
| ICOS |  | Busse et al., 2002 (VaD)^52^ |  |
| MCP-1 |  | Elahi et al., 2018 (VaD)^28^  Hu et al., 2024 (WMHs, Lacunes, PVSs)^53^ |  |
| MPO | Karel et al., 2022 (WMHs, lacunes)^51^ |  |  |
| TREM2 | Ohara et al., 2019 (VaD)^37^ |  |  |
| Other | | | |
| Complement C3 |  | Cai et al., 2025^41^ |  |
| Kallikrein |  | Dukic et al., 2016 (VaD)^46^ |  |
| MMP-2 |  | Hernandez-Guillamon et al., 2012^54^  Zhang DD et al., 2022 (WMHs, Lacunes, PVS, CMBs)^24^ |  |
| MMP-3 |  | Zhang DD et al., 2022 (WMHs, Lacunes, PVS, CMBs)^24^ |  |
| MMP-9 |  | Hernandez-Guillamon et al., 2012^54^  Hu et al., 2024 (WMHs, Lacunes, PVSs)^53^  Zhang DD et al., 2022 (WMHs, Lacunes, PVS, CMBs)^24^ |  |
| SERPINA3 | Hu et al., 2024 (WMHs)^53^ | Hu et al., 2024 (Lacunes, PVSs)^53^ |  |
| S100A8/A9 | Karel et al., 2022 (WMHs)^51^ |  |  |
| TIMP-1 |  | Hu et al., 2024 (WMHs, Lacunes, PVSs)^53^ |  |
| IgA |  | Yaqub et al., 2023 (WMHs)^55^ |  |
| IgG |  | Yaqub et al., 2023 (WMHs)^55^ |  |
| IgM |  | Yaqub et al., 2023 (WMHs)^55^ |  |

**References**

1.	Gupta A, Watkins A, Thomas P, et al. Coagulation and inflammatory markers in Alzheimer’s and vascular dementia. *Int J Clin Pract* 2005; 59: 52–57.

2.	Hao Z, Wei J, Li X, et al. Inflammation-associated D-dimer predicts neurological outcome of recent small subcortical infarct: A prospective clinical and laboratory study. *Clin Neurol Neurosurg* 2024; 237: 108126.

3.	Gallacher J, Bayer A, Lowe G, et al. Is sticky blood bad for the brain?: Hemostatic and inflammatory systems and dementia in the caerphilly prospective study. *Arterioscler Thromb Vasc Biol* 2010; 30: 599–604.

4.	Wiseman SJ, Doubal FN, Chappell FM, et al. Plasma Biomarkers of Inflammation, Endothelial Function and Hemostasis in Cerebral Small Vessel Disease. *Cerebrovascular Diseases* 2015; 40: 157–164.

5.	Aono Y, Ohkubo T, Kikuya M, et al. Plasma fibrinogen, ambulatory blood pressure, and silent cerebrovascular lesions: the Ohasama study. *Arterioscler Thromb Vasc Biol* 2007; 27: 963–968.

6.	Gao Y, Zong C, Yao Y, et al. Elevated Fibrinogen-to-Albumin Ratio Correlates with Incident Stroke in Cerebral Small Vessel Disease. *J Inflamm Res* 2024; 17: 4331–4343.

7.	Martí-Fàbregas J, Valencia C, … JP-E, et al. Fibrinogen and the amount of leukoaraiosis in patients with symptomatic small-vessel disease. *karger.comJ Martí-Fàbregas, C Valencia, J Pujol, C García-Sánchez, JL Martí-VilaltaEuropean neurology, 2002•karger.com*, https://karger.com/ene/article-abstract/48/4/185/123291 (accessed 8 February 2026).

8.	Zhang L, Tang X, Li Y, et al. Total magnetic resonance imaging of cerebral small vessel disease burden predicts dysphagia in patients with a single recent small subcortical infarct. *BMC Neurology 2021 22:1* 2022; 22: 1-.

9.	Knottnerus ILH, Govers-Riemslag JWP, Hamulyak K, et al. Endothelial activation in lacunar stroke subtypes. *Stroke* 2010; 41: 1617–1622.

10.	Kearney-Schwartz A, Rossignol P, Bracard S, et al. Vascular Structure and Function Is Correlated to Cognitive Performance and White Matter Hyperintensities in Older Hypertensive Patients With Subjective Memory Complaints. *Stroke* 2009; 40: 1229–1236.

11.	Cao L, Guo Y, Zhu Z. Effects of hyperhomocysteinemia on ischemic cerebral small vessel disease and analysis of inflammatory mechanisms. *International Journal of Neuroscience* 2021; 131: 362–369.

12.	Chen BA, Lee WJ, Meng LC, et al. Sex-specific implications of inflammation in covert cerebral small vessel disease. *BMC Neurology 2024 24:1* 2024; 24: 220-.

13.	Dufouil C, Alpérovitch A, Ducros V, et al. Homocysteine, white matter hyperintensities, and cognition in healthy elderly people. *Ann Neurol* 2003; 53: 214–221.

14.	Hassan A, Hunt BJ, O’Sullivan M, et al. Homocysteine is a risk factor for cerebral small vessel disease, acting via endothelial dysfunction. *Brain* 2004; 127: 212–219.

15.	Hogervorst E, Ribeiro HM, Molyneux A, et al. Plasma Homocysteine Levels, Cerebrovascular Risk Factors, and Cerebral White Matter Changes (Leukoaraiosis) in Patients With Alzheimer Disease. *Arch Neurol* 2002; 59: 787–793.

16.	Kloppenborg RP, Geerlings MI, Visseren FL, et al. Homocysteine and progression of generalized small-vessel disease. *Neurology* 2014; 82: 777–783.

17.	Longstreth WT, Katz R, Olson J, et al. Plasma Total Homocysteine Levels and Cranial Magnetic Resonance Imaging Findings in Elderly Persons: The Cardiovascular Health Study. *Arch Neurol* 2004; 61: 67–72.

18.	Naka H, Nomura E, Takahashi T, et al. Plasma total homocysteine levels are associated with advanced leukoaraiosis but not with asymptomatic microbleeds on T2*-weighted MRI in patients with stroke. *Eur J Neurol* 2006; 13: 261–265.

19.	Narayan SK, Firbank MJ, Saxby BK, et al. Elevated Plasma Homocysteine Is Associated with Increased Brain Atrophy Rates in Older Subjects with Mild Hypertension. *Dement Geriatr Cogn Disord* 2011; 31: 341–348.

20.	Pavlovic AM, Pekmezovic T, Obrenovic R, et al. Increased total homocysteine level is associated with clinical status and severity of white matter changes in symptomatic patients with subcortical small vessel disease. *Clin Neurol Neurosurg* 2011; 113: 711–715.

21.	Raz N, Yang Y, Dahle CL, et al. Volume of white matter hyperintensities in healthy adults: Contribution of age, vascular risk factors, and inflammation-related genetic variants. *Biochimica et Biophysica Acta (BBA) - Molecular Basis of Disease* 2012; 1822: 361–369.

22.	Staszewski J, Piusińska-Macoch R, Brodacki B, et al. IL-6, PF-4, sCD40 L, and homocysteine are associated with the radiological progression of cerebral small-vessel disease: A 2-year follow-up study. *Clin Interv Aging* 2018; 13: 1135–1141.

23.	Vermeer SE, Van Dijk EJ, Koudstaal PJ, et al. Homocysteine, silent brain infarcts, and white matter lesions: The Rotterdam Scan Study. *Ann Neurol* 2002; 51: 285–289.

24.	Zhang DD, Cao Y, Mu JY, et al. Inflammatory biomarkers and cerebral small vessel disease: a community-based cohort study. *Stroke Vasc Neurol* 2022; 7: 302–309.

25.	Gregory MA, Manuel-Apolinar L, Sánchez-Garcia S, et al. Soluble Intercellular Adhesion Molecule-1 (sICAM-1) as a Biomarker of Vascular Cognitive Impairment in Older Adults. *Dement Geriatr Cogn Disord* 2019; 47: 243–253.

26.	Engelhart MJ, Geerlings MI, Meijer J, et al. Inflammatory Proteins in Plasma and the Risk of Dementia: The Rotterdam Study. *Arch Neurol* 2004; 61: 668–672.

27.	Markus HS, Hunt B, Palmer K, et al. Markers of Endothelial and Hemostatic Activation and Progression of Cerebral White Matter Hyperintensities. *Stroke* 2005; 36: 1410–1414.

28.	Elahi FM, Casaletto KB, Altendahl M, et al. “Liquid Biopsy” of White Matter Hyperintensity in Functionally Normal Elders. *Front Aging Neurosci* 2018; 10: 411738.

29.	Hilal S, Ikram MA, Verbeek MM, et al. C-reactive protein, plasma amyloid-β levels, and their interaction with magnetic resonance imaging markers. *Stroke* 2018; 49: 2692–2698.

30.	Hoshi T, Kitagawa K, Yamagami H, et al. Relations of serum high-sensitivity C-reactive protein and interleukin-6 levels with silent brain infarction. *Stroke* 2005; 36: 768–772.

31.	Hsu PF, Pan WH, Yip BS, et al. C-Reactive Protein Predicts Incidence of Dementia in an Elderly Asian Community Cohort. *J Am Med Dir Assoc* 2017; 18: 277.e7-277.e11.

32.	Mitaki S, Nagai A, Oguro H, et al. C-reactive protein levels are associated with cerebral small vessel-related lesions. *Acta Neurol Scand* 2016; 133: 68–74.

33.	Yao H, Mizoguchi Y, Monji A, et al. Low-Grade Inflammation Is Associated with Apathy Indirectly via Deep White Matter Lesions in Community-Dwelling Older Adults: The Sefuri Study. *International Journal of Molecular Sciences 2019, Vol 20, Page 1905* 2019; 20: 1905.

34.	Helmy AA, Abdel Naseer MM, El Shafie S, et al. Role of Interleukin 6 and Alpha-Globulins in Differentiating Alzheimer and Vascular Dementias. *Neurodegener Dis* 2012; 9: 81–86.

35.	Huang CJ, Zhou X, Yuan X, et al. Contribution of Inflammation and Hypoperfusion to White Matter Hyperintensities-Related Cognitive Impairment. *Front Neurol* 2022; 12: 786840.

36.	Miwa K, Okazaki S, Sakaguchi M, et al. Interleukin-6, interleukin-6 receptor gene variant, small-vessel disease and incident dementia. *Eur J Neurol* 2016; 23: 656–663.

37.	Ohara T, Hata J, Tanaka M, et al. Serum Soluble Triggering Receptor Expressed on Myeloid Cells 2 as a Biomarker for Incident Dementia: The Hisayama Study. *Ann Neurol* 2019; 85: 47–58.

38.	Satizabal CL, Zhu YC, Dufouil C, et al. Inflammatory proteins and the severity of dilated Virchow-Robin spaces in the elderly. *Journal of Alzheimer’s Disease* 2013; 33: 323–328.

39.	Wada M, Nagasawa H, Kurita K, et al. Cerebral small vessel disease and C-reactive protein: Results of a cross-sectional study in community-based Japanese elderly. *J Neurol Sci* 2008; 264: 43–49.

40.	Bai L, Yan H, Wang Z, et al. Expression of inflammatory cytokines in CADASIL and their associations with clinical and neuroimaging features. *Front Immunol* 2025; 16: 1650847.

41.	Cai H, Zhao T, Pang Y, et al. Systemic inflammatory markers in ageing, Alzheimer’s disease and other dementias. *Brain* 2025; 148: 480–492.

42.	Jefferson AL, Massaro JM, Wolf PA, et al. Inflammatory biomarkers are associated with total brain volume: The Framingham Heart Study. *Neurology* 2007; 68: 1032–1038.

43.	Zuliani G, Ranzini M, Guerra G, et al. Plasma cytokines profile in older subjects with late onset Alzheimer’s disease or vascular dementia. *J Psychiatr Res* 2007; 41: 686–693.

44.	Hanning U, Roesler A, Peters A, et al. Structural brain changes and all-cause mortality in the elderly population—the mediating role of inflammation. *AGE 2016 38:5* 2016; 38: 455–464.

45.	Zuliani G, Guerra G, Ranzini M, et al. High interleukin-6 plasma levels are associated with functional impairment in older patients with Vascular dementia. *Int J Geriatr Psychiatry* 2007; 22: 305–311.

46.	Dukic L, Simundic AM, Martinic-Popovic I, et al. The role of human kallikrein 6, clusterin and adiponectin as potential blood biomarkers of dementia. *Clin Biochem* 2016; 49: 213–218.

47.	Liu D, Chi LJ. Association Between VISTA and Vascular Cognitive Impairment in Older Chinese Adults: A Cross-Sectional Study. *Clin Interv Aging* 2024; 19: 1939–1949.

48.	Oberheiden T, Blahak C, Nguyen XD, et al. Activation of platelets and cellular coagulation in cerebral small-vessel disease. *Blood Coagulation and Fibrinolysis* 2010; 21: 729–735.

49.	Altendahl M, Maillard P, Harvey D, et al. An IL-18-centered inflammatory network as a biomarker for cerebral white matter injury. *PLoS One* 2020; 15: e0227835.

50.	Malaguarnera L, Motta M, Di Rosa M, et al. Interleukin-18 and transforming growth factor-beta 1 plasma levels in Alzheimer’s disease and vascular dementia. *Neuropathology* 2006; 26: 307–312.

51.	Karel MFA, Roosen MGCH, Tullemans BME, et al. Characterization of cerebral small vessel disease by neutrophil and platelet activation markers using artificial intelligence. *J Neuroimmunol* 2022; 367: 577863.

52.	Busse S, von Hoff F, Michler E, et al. Altered expression of costimulatory molecules in dementias. *European Archives of Psychiatry and Clinical Neuroscience 2021 272:5* 2021; 272: 807–815.

53.	Hu X, Xiao ZS, Shen YQ, et al. SERPINA3: A novel inflammatory biomarker associated with cerebral small vessel disease burden in ischemic stroke. *CNS Neurosci Ther* 2024; 30: e14472.

54.	Hernandez-Guillamon M, Martinez-Saez E, Delgado P, et al. MMP-2/MMP-9 plasma level and brain expression in cerebral amyloid angiopathy-associated hemorrhagic stroke. *Brain Pathology* 2012; 22: 133–141.

55.	Yaqub A, Khan SR, Vernooij MW, et al. Serum immunoglobulins and biomarkers of dementia: a population-based study. *Alzheimer’s Research & Therapy 2023 15:1* 2023; 15: 194-.
